# 124 Using Co-Design Methods to Develop a Theory and Evidence-Based Mother-Daughter mHealth Intervention Prototype Targeting Physical Activity in Pre-Teen Girls

**DOI:** 10.1093/eurpub/ckae114.120

**Published:** 2024-09-26

**Authors:** James Matthews, Carol Brennan, Grainne O’Donoghue, Alison Keogh, Ryan Rhodes

**Affiliations:** University College Dublin, Dublin, Ireland; University College Dublin, Dublin, Ireland; University College Dublin, Dublin, Ireland; Trinity College, Dublin, Ireland; University of Victoria, Victoria, Canada

## Abstract

**Purpose:**

Pre-teen girls of lower socio-economic position are at increased risk of physical inactivity. Parental support, particularly mothers, is positively correlated with girls’ physical activity levels. Consequently, family-based interventions are recognised as a promising approach to improve young people’s physical activity. However, the effects of these interventions on girls’ physical activity are often inconsistent, with calls for more rigorous, theory based and co-designed family-based interventions to promote physical activity in this cohort. Therefore, the aim of this study was to use co-design methods to develop a theory and evidence- based mother daughter mHealth intervention prototype targeting physical activity in pre-teen girls.

**Method:**

The intervention prototype was developed in accordance with the UK Medical Research Council framework, the Behaviour Change Wheel, the Theoretical Domains Framework, and the Behaviour Change Techniques Taxonomy V1. The co-design process incorporated three phases, (i) behavioural analysis, (ii) the selection of intervention components, and (iii) refinement of the intervention prototype. Across these phases, there were workshops with pre-teen girls (n = 10); mothers of pre-teen girls (n = 9), and primary school teachers (n = 6), with further input from an expert advisory group.

**Results:**

This three-phase co-design process resulted in the development of a theory-based intervention which targeted two behaviours, (i) mothers’ engagement in a range of supportive behaviours for their daughters’ physical activity, and (ii) daughters’ physical activity behaviour. Formative research identified eleven theoretical domains to be targeted as part of the intervention (e.g., knowledge, skills, and beliefs about capabilities). These were to be targeted by six intervention functions (e.g., education, persuasion, modelling), and 28 behaviours change techniques (e.g., goal setting, self-monitoring). The co-design process resulted in a mobile app being chosen as the mode of delivery for the intervention.

**Conclusion:**

This paper offers a rich description of using co-design methods to develop a mother-daughter mHealth intervention prototype, that is ready for feasibility and acceptability testing. The use of theoretical frameworks in the co-design process provided a robust and transparent foundation on which to develop the prototype, enabling the evaluation of potential pathways for behaviour change.

